# Microbial Community, Co-Occurrence Network Relationship and Fermentation Lignocellulose Characteristics of *Broussonetia papyrifera* Ensiled with Wheat Bran

**DOI:** 10.3390/microorganisms10102015

**Published:** 2022-10-12

**Authors:** Wenbo Wang, Yanshun Nie, Hua Tian, Xiaoyan Quan, Jialin Li, Qiuli Shan, Hongmei Li, Yichao Cai, Shangjun Ning, Ramon Santos Bermudez, Wenxing He

**Affiliations:** 1 School of Biological Science and Technology, University of Jinan, Jinan 250022, China; 2 Fengtang Ecological Agriculture Technology Research and Development (Shandong) Co., Ltd., Taian 271400, China; 3 Faculty of Agricultural Sciences, Luis Vargas Torres de Esmeraldas University of Technology, Esmeraldas 080103, Ecuador

**Keywords:** *Broussonetia papyrifera* silage, fermentation lignocellulose characteristic, microbial community, co-occurrence network, metabolism function

## Abstract

*Broussonetia papyrifera* has a high lignocellulose content leading to poor palatability and low digestion rate of ruminants. Thus, dynamic profiles of fermentation lignocellulose characteristics, microbial community structure, potential function, and interspecific relationships of *B. papyrifera* mixing with wheat bran in different ratios: 100:0 (BP100), 90:10 (BP90), 80:20 (BP80), and 65:35 (BP65) were investigated on ensiling days 5, 15, 30, and 50. The results showed that adding bran increased the degradation rate of hemicellulose, neutral detergent fiber, and the activities of filter paper cellulase, endoglucanase, acid protease, and neutral protease, especially in the ratio of 65:35. *Lactobacillus*, *Pediococcus*, and *Weissella* genus bacteria were the dominant genera in silage fermentation, and *Pediococcus* and *Weissella* genus bacteria regulated the process of silage fermentation. Compared with monospecific *B. papyrifera* silage, adding bran significantly increased the abundance of *Weissella* sp., and improved bacterial fermentation potential in BP65 (*p* < 0.05). Distance-based redundancy analysis showed that lactic acid bacteria (LAB) were significantly positive correlated with most lignocellulose content and degrading enzymes activities, while *Monascus* sp. and *Syncephalastrum* sp. were opposite (*p* < 0.05). Co-occurrence network analysis indicated that there were significant differences in microbial networks among different mixing ratios of *B. papyrifera* silage prepared with bran. There was a more complex, highly diverse and less competitive co-occurrence network in BP65, which was helpful to silage fermentation. In conclusion, *B. papyrifera* ensiled with bran improved the microbial community structure and the interspecific relationship and reduced the content of lignocellulose.

## 1. Introduction

With the continuous improvement of the quality of life, people’s demand and quality requirements for livestock products are also increasing. The production of conventional raw feed materials, such as corn and soybean, does not meet the development of animal husbandry. *Broussonetia papyrifera* is widely distributed in China, Thailand, and the United States because of its strong stress resistance, good adaptability and strong growth ability [1]. *B. papyrifera*, as a high-quality unconventional high protein; phenols and flavonoids have become a research hotspot in the feed field [2]. As a woody feed, *B. papyrifera* has a high content of lignocellulose, which leads to poor palatability, low digestion and utilization rate of ruminants [3]. Each silage material has its particularity; only by fully understanding the succession of the microbial community structure, metabolic function and interspecific relationship can we regulate the fermentation process and improve the quality of silage fermentation.

Ensiling has been regarded as an effective way of preserving a fresh forage due to its maximum storage duration, good palatability, and high nutrition [4]. Utilizing silage is an ideal method for maintaining forage, and it can help overcome the discrepancy between yearlong livestock production and the seasonal imbalance of available forage [5]. However, the natural fermentation of high-moisture *B. papyrifera* makes it difficult to produce high-quality silage, and butyric acid fermentation, nutrient loss and the reproduction of deleterious bacteria easily occur during ensiling, which leading to the reduction in feed quality [6,7]. Generally, the mixing of high-moisture forages with dry crop byproduct is an effective way to solve the problem of high-moisture silage. Wheat bran is an abundant agricultural by-product that is widely used as a feed resource throughout the world. Ensilage of high-moisture *B. papyrifera* with dry bran can achieve acceptable silage fermentation by reducing the moisture content and removing some of the negative characteristics of *B. papyrifera*, such as poor fermentation and low digestibility or taste [8]. At present, studies on mixed silage have been reported, and most of the relevant studies focus on the relationship between bacterial community composition, diversity and silage fermentation characteristics (including lactic acid, butyric acid and dry matter and water-soluble carbohydrates) [9,10]. As *B. papyrifera* was a woody plant, the content of lignocellulose in its stems and leaves was high, which reduced its nutritional value as silage and limits its large-scale application. Fermentation of silage lignocellulose with additives that were easier to ferment was a very good solution. The degradation and utilization of lignocellulose usually included the following ways: acid and alkali hydrolysis, enzymatic treatment and pyrolysis [11]. Unfortunately, chemical hydrolysis produced many harmful metabolites, and enzymatic treatment was very expensive. Recent research showed that the intestinal microbiota of mealworm larvae could degrade celluloses, and this method provided a new insight for effective utilization of lignocellulose [12].

It is reported that the analysis of bacterial and fungal community succession will help us to understand the relationship between fermentation characteristic of silage and microbial community composition [13,14]. It was found that lactic acid bacteria played a leading role in silage fermentation. Therefore, most studies focused on the relationship between bacterial community characteristics and fermentation quality [9,15]. However, the study found that the function of fungi in silage fermentation could not be ignored [16]. To the best of our knowledge, few studies have focused on the succession characteristics of fungal community, especially the study on the relationship between bacteria and fungi during the silage fermentation are less. Hence, the aims of our study were to investigate the effects of *B. papyrifera* silage fermentation lignocellulose characteristics, bacterial and fungal community composition, metabolism functions and interspecific relationship when ensiled with bran at different ratios and specify the optimal mixing proportion. We hypothesized that different mixing proportions affected silage fermentation lignocellulose characteristics by changing the succession and interspecific relationship of the bacterial and fungal community.

## 2. Materials and Methods

### 2.1. Silage Preparation

*B. papyrifera* were harvested on the 7 June 2021 from the 66 hectares planting base of Fengtang Ecological Agriculture Co., Ltd. (Taian, China). The silage materials were chopped into an approximate length of 1 cm with an automatic forage chopper and immediately taken to the laboratory. *B. papyrifera* (BP) and wheat bran were mixed at ratios of 100:0 (BP100), 90:10 (BP90), 80:20 (BP80), 65:35 (BP65). Each sample (approximately 200 g) was packed into vacuum-sealed polyethylene plastic bags (dimensions 170 mm × 250 mm, Xilong Packing Co., Ltd., Hebei, China) after full mixing and ensiled for 5, 15, 30, and 50 d at ambient temperature. Silage quality was classified according to appearance, smell and chemical composition [17]. Three bags of each treatment were selected at each sampling time to analyze the dynamic changes of silage lignocellulose characteristics, enzyme activities, bacterial and fungal community composition and potential function.

### 2.2. Silage Chemical Composition and Enzyme Activity Assays

The silage samples were dried at 65 °C for 72 h to constant weight. Then the dry samples were ground through a 1 mm Willy mill for chemical analysis. The content of crude protein (CP) was determined by the Kjeldahl method with automatic kjeldahl apparatus (NKB3000) [18]. The content of cellulose, hemicellulose, lignin, acid detergent fiber (ADF) and neutral detergent fiber (NDF) in silage were analyzed according to Van Soest et al. [19].

The activities of filter paper cellulase, endoglucanase, exoglucanase, β-glucosidase, acid and neutral protease were determined. All spectrophotometric measurements were made with a microplate photometer. Dissolve 10 g fermented feed in 90 mL distilled water, stand for 2 h, centrifuge at 4000 rpm for 10 min, and take the supernatant as the crude enzyme solution.

Filter paper cellulase activity was measured using Whatman no.1 filter paper by the dinitrosalicylic acid method [20]. The activity of acid and neutral protease was determined using the Folin–Ciocalteu method [21]. The activities of enzyme, endoglucanase, and exoglucanase were estimated using the 3, 5-dinitrosalicylic acid (DNS) method [22]. The activity of β-glucosidase was measured by the p-nitrophenol matrix method [23].

### 2.3. Microbial Community Analysis

#### 2.3.1. Total DNA Extraction

According to the manufacturer’s instructions, total microbial community genomic DNA was extracted from 200 mg of silage samples using the MP FastDNA SPIN Kit for Soil. Duplicate DNA extractions were performed for each sample and pooled. The quality of the extracted DNA was tested on 1% agarose gels and NanoDrop 2000 UV-vis spectrophotometer (Thermo Scientific, Wilmington, NC, USA).

#### 2.3.2. High-Throughput Sequencing of Bacterial 16SrDNA and Fungal ITS Region

The hypervariable V3–V4 region of the bacterial 16S rRNA gene was amplified with the primer pairs 799F (AACMGGATTAGATACCCKG) and 1193R (ACGTCATCCCCACCTTCC) by an ABI GeneAmp^®^ 9700 polymerase chain reaction (PCR) thermocycler (ABI, CA, USA). The primers ITS3/ITS4 were amplified with the primer pairs ITS3F (GCATCGATGAAGAACGCAGC) and ITS4R (TCCTCCGCTTATTGATATGC). The PCR amplification conditions were as follows: 95 °C for 3 min; 27 cycles of 95 °C for 30 s, 55 °C for 30 s, and 72 °C for 45 s; 72 °C for 10 min; and indefinite hold at 4 °C upon completion. The PCR condition was the same except that the number of fungal PCR cycles was 35. The PCR mixtures contained 4 μL of 5 × TransSTART FastPfu buffer, 2 μL of 2.5 mM dNTPs, 0.8 μL of forward primer (5 μM), 0.8 μL of reverse primer (5 μM), 0.4 μL of TransSTART FastPfu DNA Polymerase, 10 ng of template DNA, and sufficient ddH2O to achieve a final volume of 20 μL. PCR reactions were carried out in triplicate. The PCR product was extracted from 2% agarose gel and purified using the AxyPrep DNA Gel Extraction Kit (Axygen Biosciences, Union City, CA, USA), in accordance with the manufacturer’s instructions, then quantified using a Quantus™ Fluorometer (Promega, CA, USA).

#### 2.3.3. Bioinformatics Analysis

Paired-end reads were obtained by illumine MiSeq-PE300 sequencing platform, then spliced on the basis of their overlapping relationships. The Fastp software (https://github.com/OpenGene/fastp, version 0.20.0, accessed on 11 May 2022) and FLASH software (http://www.cbcb.umd.edu/software/flash, version 1.2.7, accessed on 11 May 2022) were used to control the quality of the original sequencing sequence and splice sequence. The raw data generated by sequencing in this study were publicly available in the NCBI Sequence Read Achieve (SRA) database with an accession number PRJNA868905. After differentiation of the samples, the operational taxon (OTU) clustering analysis and species classification analysis were carried out; multiple diversity index analysis and sequencing depth detection were conducted for OTU. In order to obtain species classification information about each OTU, the RDP classifier Bayesian algorithm was used to analyze representative OTU sequences at 97% similarity level. The bacterial and fungal taxonomy of each OTU representative sequence were analyzed against the 16S rRNA database (Silva v138) and ITS database (unite 8.0) using confidence threshold of 0.7. QIIME software (version 1.9.1) was used to analyze abundance information of various taxonomic levels of microbial communities. Then various in-depth statistical and visual analyses were performed to determine the community composition and potential functions. FAPROTAX was used to predict the functional potential of bacteria (http://www.loucalab.com/archive/FAPROTAX/, accessed on 1 June 2022) [24]. Functions of fungal communities were classified and analyzed by FUNGuild (http:// https://github.com/UMNFuN/FUNGuild, accessed on 11 June 2022), with the fungi divided into pathotrophs, symbiotrophs, and saprotrophs [25].

#### 2.3.4. Statistical Analysis

The effect of mixing *B. papyrifera* with different wheat bran ratios on the silage chemical composition and enzyme activity were performed by SPSS software (SPSS Inc., Chicago, IL, USA). The *p*-value of < 0.05, 0.01 and 0.001 were considered statistically significant. If the data did not meet normality assumption, the values were converted to logarithmic and/or square root before statistical analysis. Statistical differences among means were evaluated using Tukey’s multiple comparison. Repeated measures analysis of variance (RMANOVA) was used to test significant differences among mixing proportions and fermentation time in the contents of silage chemical components, enzyme activity, and relative abundances of function genes [26]. The Spearman’s correlation heatmaps between bacterial and fungal groups were constructed via R software (Version 3.3.1) pheatmap package [27]. The Kruskal-Wallis H test was used to analyze the difference of functional bacterial community categories among different samples [28]. Distance-based redundancy analysis (db-RDA) was performed using the vegan package in R software to investigate the relationships among microbial composition, enzyme activity, and silage chemical components content [29]. A co-occurrence network was constructed using 12 samples of each proportion silage samples. Co-occurrence network analyses of bacterial and fungal communities were conducted using the Python package ‘networkx’ [30]. To simplify pairwise comparisons and reduce the complexity of co-occurrence network, only the genera with relative abundance of top 50 were selected for network analyses [31]. The relationships with strong positive (r > 0.7) and strong negative (r < −0.7) were shown in the network diagrams. The species with a high mean degree, high closeness centrality, and high betweenness centrality were regard as keystone taxa [32].

## 3. Results and Discussion

### 3.1. Dynamic Analysis of Chemical Composition and Enzyme Activity during Silage Fermentation

**Figure 1:**
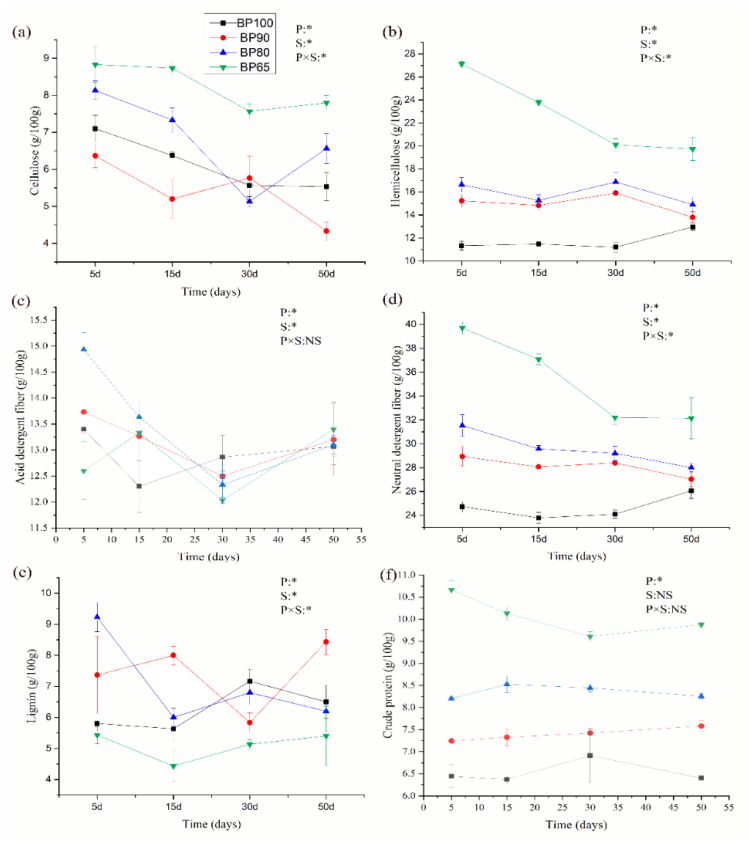
Dynamics of chemical composition including (**a**) cellulose, (**b**) hemicellulose, (**c**) acid detergent fiber, (**d**) neutral detergent fiber, (**e**) lignin, and (**f**) crude protein during silage fermentation. RMANOVA results were reported. P: mixing proportion; S: sampling time; NS, not significant (*p* > 0.05); * *p* < 0.05.

The dynamics of the chemical composition of *B. papyrifera* and bran at different mixing ratio silages were presented in Figure 1. RMANOVA results showed that the content of cellulose, hemicellulose, lignin, acid detergent fiber and neutral detergent fiber were significantly affected by the mixing ratio and fermentation time (*p* < 0.05). The content of crude protein was not significantly affected by fermentation time (*p* > 0.05). This result was consistent with the findings of Der Bedrosian et al. [33]. One possible reason was that the protease in silage mainly came from mold, which was usually inhibited by anaerobic environment and lactic acid bacteria during silage fermentation [34]. During 5 to 50 days of silage fermentation, it was found that the highest degradation rate of cellulose was found in BP90 (31.94%); hemicellulose was found in BP65 (27.27%); lignin was found in BP80 (32.85%); neutral detergent fiber was found in BP65 (19.06%); crude protein was found in BP65 (7.41%); and acid detergent fiber was found in BP80 (12.28%). In the composition of silage raw materials, the content of hemicellulose and neutral detergent fiber were higher. These results were consistent with the research data of Dong et al. [9]. It was found that different mixing proportion of silage materials would significantly affect the fermentation lignocellulose characteristics, especially for the content of neutral detergent fiber and acid detergent lignin [35]. This study found that compared with other mixing ratios, there were greater degradation rates of hemicellulose (27.27%) and neutral detergent fiber (19.06%) in BP65. From the above, adding bran to silage reduced the content of cellulose, hemicellulose, crude protein, acid detergent fiber and neutral detergent fiber over the 50 d silage time. Therefore, adding bran may potentially contribute to enriching the nutritional composition and improve the feeding effect.

**Figure 2:**
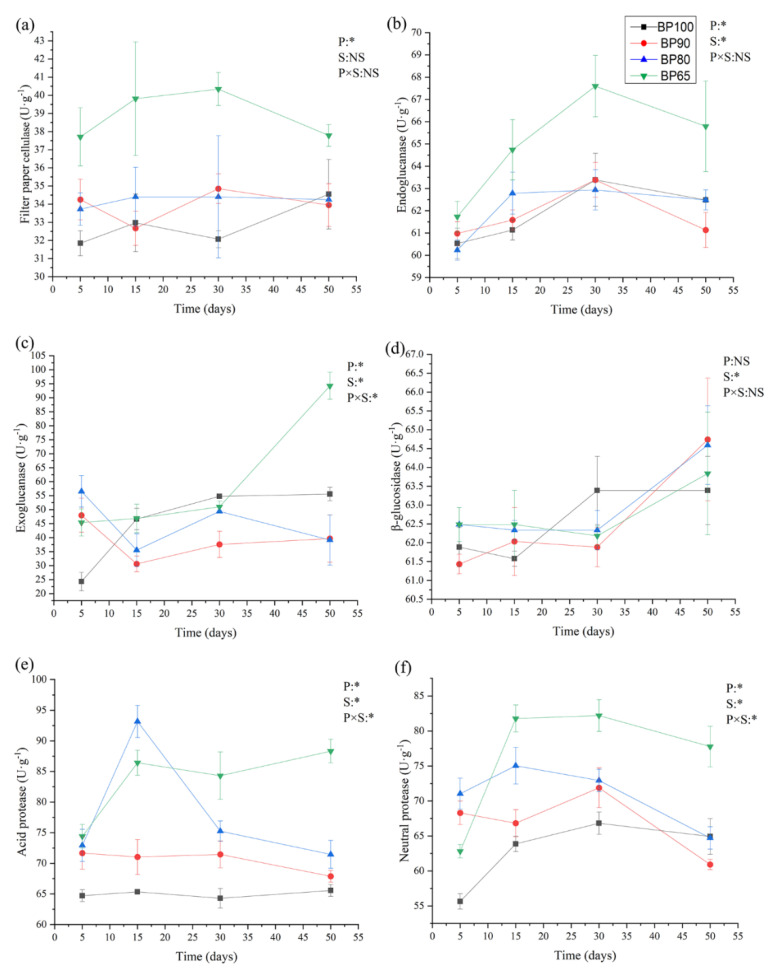
Dynamics of enzyme activities including (**a**) filter paper cellulase, (**b**) endoglucanase, (**c**) exoglucanase, (**d**) β-glucosidase, (**e**) acid protease, and (**f**) neutral protease during silage fermentation. RMANOVA results were reported. P: mixing proportion; S: sampling time; NS, not significant (*p* < 0.05); * *p* < 0.05.

As shown in Figure 2, the activities of filter paper cellulase, endoglucanase, exoglucanase, acid protease, and neutral protease were significantly affected by mixing ratio (*p* < 0.05). Except for filter paper, the activities of the other seven enzymes were significantly affected by silage fermentation time (*p* < 0.05). With the increase in fermentation time, the activity of β-glucosidase significantly increased (*p* < 0.05) (Figure 2d). The activities of filter paper cellulase, endoglucanase, acid and neutral protease in BP65 were significantly higher than BP100 (Figure 2a,b). It was reported that filter paper cellulase, and endoglucanase played an important role in silage fermentation [20]. The appropriate amount of protease could effectively improve ruminal in vitro star digestibility, and did not have a negative impact on other characteristics of silage [36]. This indicated that there was better fermentation effect in BP65.

### 3.2. Composition Succession Characteristics of Bacterial and Fungal Community

**Figure 3:**
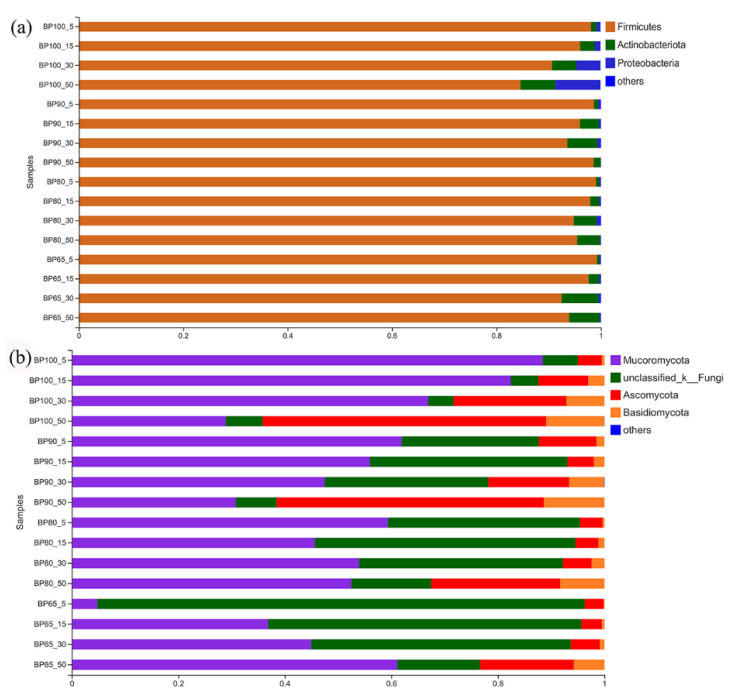
The relative abundances of bacterial (**a**) and fungal community (**b**) structure at phylum levels.

The predominant bacterial phyla in all samples were Firmicutes (95%), followed by Actinobacteriota (3%) and Proteobacteria (1%) (Figure 3). Low pH and anaerobic environmental conditions may be the main driving force for shaping this community structure [37]. With the progress of silage fermentation, the relative abundance of Firmicutes was gradually decreased, while Actinobacteriota increased. This result was the same as that of Yin et al. [38]. Among different mixing ratios, the relative abundance of Proteobacteria in BP100 was significantly higher. Proteobacteria play a significant role in organic matter degradation during anaerobic digestion, and some genera can use lactic acid leading to nutrient loss [39,40]. The predominant fungal phyla in all samples were Mucoromycota (51%), followed by unclassified_Fungi (29%) and Ascomycota (14%). With the progress of silage fermentation, the relative abundance of Ascomycota and Basidiomycota significantly increased (*p* < 0.05). The relative abundance of Mucoromycota in BP65 showed opposite trend than other samples during silage fermentation time. This may be due to the mixture of *B. papyrifera* and bran changing the microbial community structure, resulting in changes in the succession of microbial community [9].

**Figure 4:**
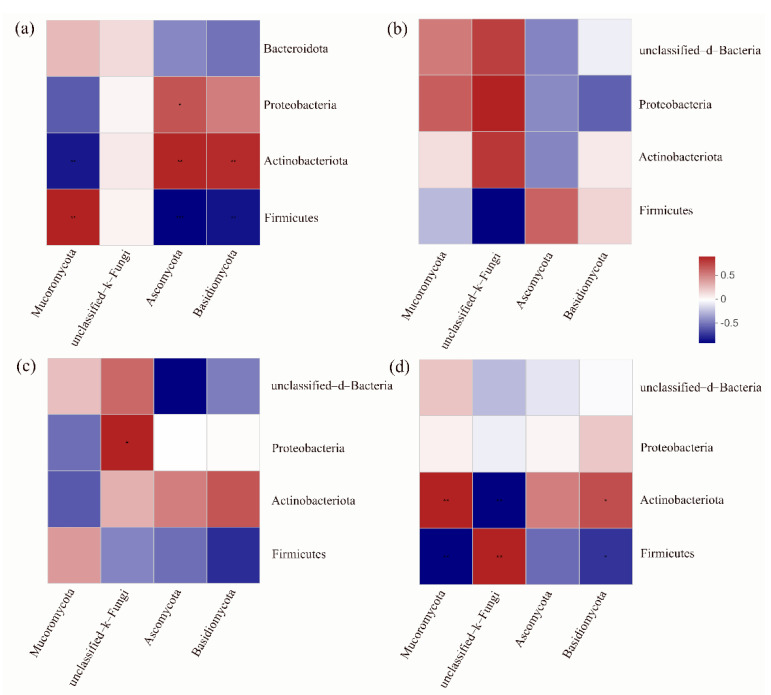
Correlation analysis of bacterial and fungal community compositions at phylum levels. (**a**) BP100; (**b**) BP90; (**c**) BP80; (**d**) BP65. * *p* < 0.05, ** *p* < 0.01.

The Spearman’s correlation heatmaps (Figure 4) were used to elucidate relationships among different samples. The result showed that there were differences in the correlation between bacterial and fungal phyla in BP100 and BP65. There was a significant negative correlation between Firmicutes and Mucoromycota in BP65, while it was opposite in BP100. It has been found that Mucoromycota was the common fungal pathogen detected in deteriorated silage [41]. Hence, the interspecific relationships of bacterial and fungal phyla in BP65 were helpful to reduce the risk of silage fermentation deterioration.

**Figure 5:**
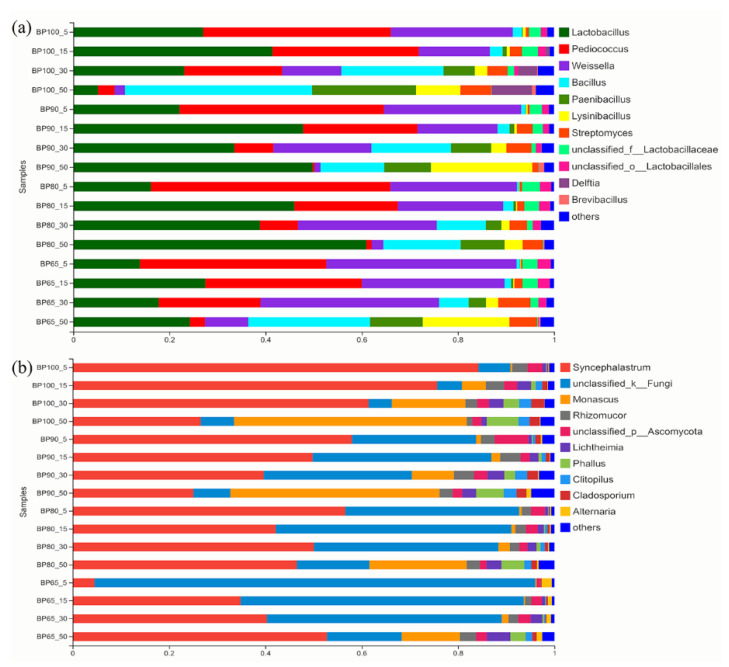
The relative abundances of bacterial (**a**) and fungal community (**b**) structure at genus levels.

At the genus level of bacteria, the main genera in all samples were *Lactobacillus* (31%), followed by *Pediococcus* (21%) and *Weissella* (20%) genus bacteria (Figure 5). As reported, *Lactobacillus* sp., *Pediococcus* sp., and *Weissella* sp. were important silage fermentation functional flora [42]. At days 30 and 50 of silage fermentation, the total abundance of *Lactobacillus* sp., *Pediococcus* sp., and *Weissella* sp. were higher in BP65, BP80 and BP90 than in BP100. This indicated that *B. papyrifera* mixing with bran was more conducive to silage fermentation. Moreover, the relative abundance of *Weissella* sp. in BP65 was significantly higher than that of BP100 (*p* < 0.05). The study found that *Weissella* sp. have strong inhibitory effect against molds [43]. While the relative abundance of *Bacillus* sp. was significantly higher in BP100 than the other mixing ratio samples on days 50 (*p* < 0.05). Bacillus was related to the aerobic degradation of silage, and its increased abundance meant that the sample was more prone to deterioration [44]. With the progress of fermentation, the relative abundance of *Bacillus* sp. and *Paenibacillus* sp. increased significantly (*p* < 0.05). It was reported that *Bacillus* sp. and *Paenibacillus* sp. would compete with lactic acid bacteria for substrates to inhibit the growth of lactic acid bacteria [45]. Therefore, it was observed in our study that with the extension of silage fermentation time, the total abundance of *Lactobacillus* sp., *Pediococcus* sp., and *Weissella* sp. were significantly decreased (*p* < 0.05). In addition to BP100, the abundance of *Lactobacillus* sp. gradually increased in other samples during 50 d silage fermentation. The study found that most of the early bacteria would be completely replaced by *Lactobacillus* sp. after day 49 of fermentation [46]. It can be speculated that with the further extension of fermentation time, *Lactobacillus* sp. will become the dominant genus.

At the genus level of fungi, the main genera in all samples were *Syncephalastrum* (47%), followed by *unclassified_Fungi* (30%) and *Monascus* (10%) genus fungi. It was reported that *Monascus* sp. and *Syncephalastrum* sp. might cause aerobic spoilage in silage, and *Syncephalastrum* sp. could inhibit bacterial growth [40,47]. With the progress of silage fermentation, the relative abundance of *Syncephalastrum* sp. showed the opposite trend between BP65 and the other mixing ratio. The relative abundance of *Monascus* sp. significantly increased in all samples (*p* < 0.05). According to the reports, *Monascus* sp.could grow rapidly due to its resistance to heat, lactic acid and survive under reduced oxygen levels [48,49]. It was this characteristic of *Monascus* sp. that enabled it to become a dominant genus in the late stage of fermentation. The relative abundance of *Monascus* sp. were significantly lower in BP65, and BP80 than in BP100. This may be due to the higher abundance of *Weissella* sp. in BP65 and BP80 inhibited the growth and reproduction of *Monascus* sp.

**Figure 6:**
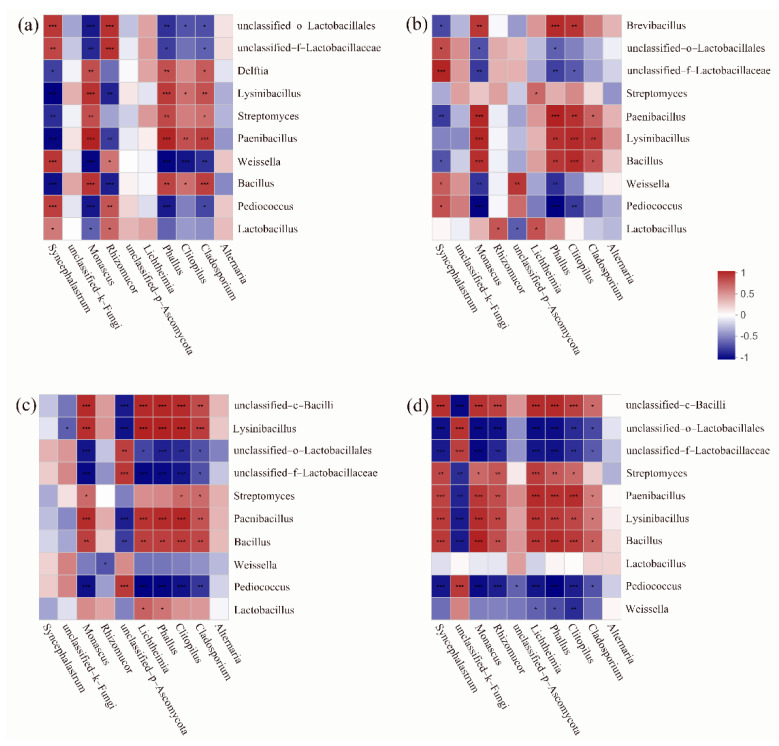
Correlation analysis of bacterial and fungal community compositions at genus levels. (**a**) BP100; (**b**) BP90; (**c**) BP80; (**d**) BP65. * *p* < 0.05, ** *p* < 0.01, *** *p* < 0.001.

The Spearman’s correlation heatmaps (Figure 6) were used to elucidate relationships among different samples. The result showed that there were differences in the correlation between bacterial and fungal genera in different samples. It is worth noting that in BP65, except *unclassified_Fungi*, *Pediococcus* sp. and *Weissella* sp. were negatively correlated with other fungal genera. This indicated that *Pediococcus* sp. and *Weissella* sp. were the dominant flora in microbial community, and would regulate the process of silage fermentation.

### 3.3. Functional Succession Characteristics of Bacterial and Fungal Community

**Figure 7:**
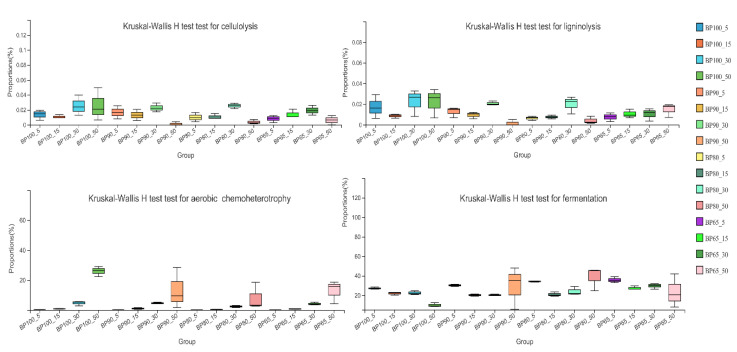
Functional bacterial community categories inferred by FAPROTAX function prediction.

The RMANOVA results showed that bacterial cellulolysis function, ligninolysis function, aerobic chemoheterotrophy function and fermentation function were significantly affected by mixing ratio and fermentation time (*p* < 0.05) (Figure 7). With the extension of fermentation time, the mean proportions of cellulolysis function and ligninolysis function increased first and then decreased, fermentation function gradually decreased. The mean proportions of aerobic chemoheterotrophy function increased during silage fermentation. Especially after 30 days of silage, the mean proportions of aerobic chemoheterotrophy function significantly increased. The reason for this change may be related to the significant decrease in total abundance of *Lactobacillus* sp., *Pediococcus* sp., and *Weissella* sp. after 30 days of silage. It has been reported that there were negative correlations between anaerobic and aerobic microbial groups [8], so the decrease in abundance of *Lactobacillus* sp., *Pediococcus* sp., and *Weissella* sp. may be caused by interspecific competition among *Bacillus* sp., *Syncephalastrum* sp. and *Monascus* sp. The result meant that with the progress of silage, there were more silage substrates consuming by aerobic microorganism. In general, the suitable time for silage fermentation should be around the 30th day. Dong et al. [9] found that lactic acid bacteria population and lactic acid content reached a peak on the 30th day during the 60 days silage fermentation. The result confirmed that the abundance of lactic acid bacteria and silage fermentation function were closely related.

The bacterial fermentation potential increased when *B. papyrifera* and bran were mixed. Especially on the 5th, 15th and 30th day of silage fermentation, the mean proportions of bacterial fermentation function BP65 were significantly higher than in BP100 (*p* < 0.05). Overall, the mixture of *B. papyrifera* and bran at the ratio of 65:35 would produce better feed quality. A possible reason was that the protein content of *B. papyrifera* leaves was high, and the content of water-soluble carbohydrates was low, which made silage fermentation difficult. However, the content of water-soluble carbohydrates in bran was higher, and the nutrient composition was more reasonable after mixing in an appropriate proportion, which was more conducive to the growth of silage functional flora [50].

**Figure 8:**
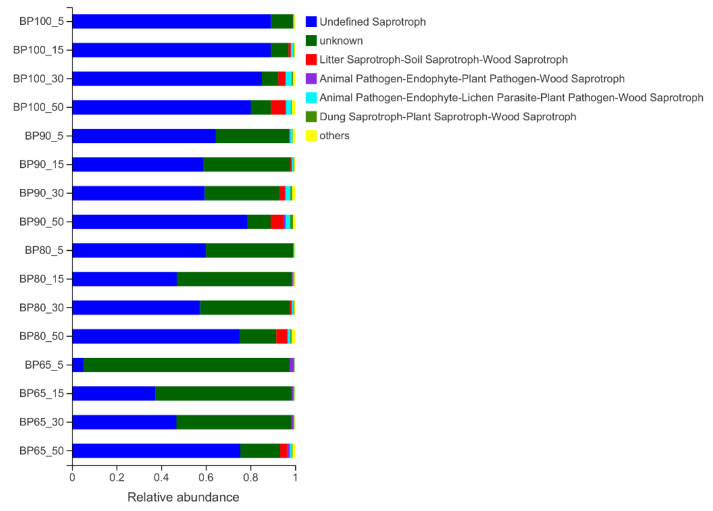
Variations in fungal functional groups compositions inferred by FUNGuild.

Fungal functions were classified by FUNGuild (Figure 8). The results showed that with the increase in silage fermentation time, the proportions of saprotroph in BP100 gradually decreased. However, the proportions of saprotroph in BP90, and BP65 significantly increased (*p* < 0.05). This may be caused by competitive interactions between fungal and bacterial groups, for example, the relative abundance of *Lactobacillus* sp., *Weissella* sp. and *Pediococcus* sp. decreased with the progress of silage fermentation [51]. The saprotroph was usually bad for silage fermentation, and lead to the spoilage of the substrate and reduce the nutritional quality of silage [52]. It is worth noting that the proportions of animal pathogen gradually increased during silage fermentation time in all samples. Saprophytic fungi usually produce mycotoxins, resulting in increased pathogenicity of the flora [53]. Hence, excessive fermentation could reduce the quality of feed and increase the risk of disease of feeding animals. While the proportions of animal pathogen were significant lower in BP65 than in BP100 (*p* < 0.05). In general, the mixture of *B. papyrifera* and bran reduced the proportion of saprophytic fungi and animal pathogenic fungi, and BP65 was the better mixture ratio.

### 3.4. Correlation Analysis of Environmental Factors

**Figure 9:**
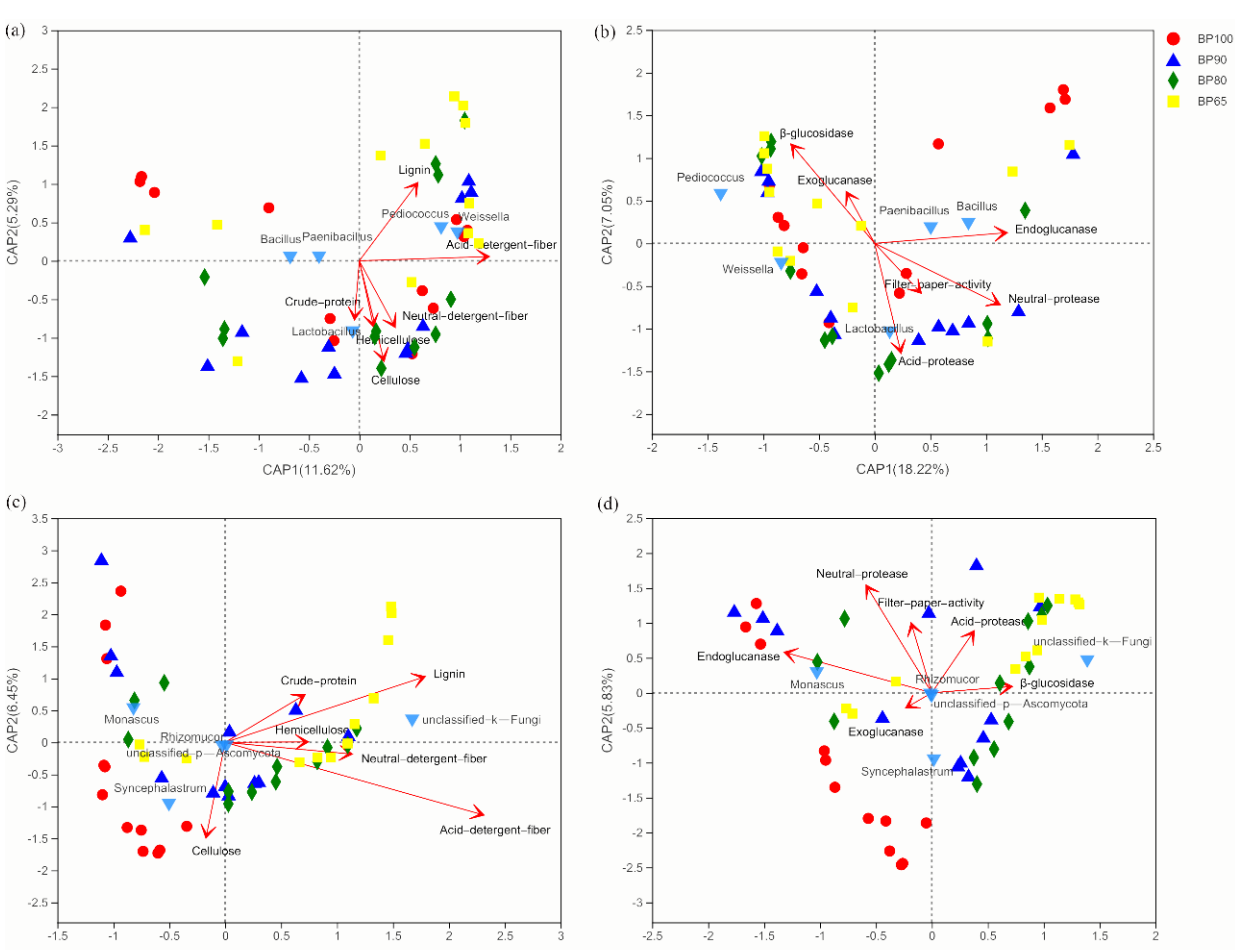
Distance-based redundancy analysis (db-RDA) to show correlation among microbial community, silage chemical composition and enzyme activity in different samples. (**a**) Bacterial genera and chemical composition; (**b**) bacterial genera and enzymatic activity; (**c**) fungal genera and chemical composition; (**d**) fungal genera and enzymatic activity.

Distance-based redundancy analysis showed that the bacterial community compositions were significantly correlated with cellulose, hemicellulose, lignin, acid detergent fiber and neutral detergent fiber (R^2^ = 0.36, *p* = 0.001; R^2^ = 0.15, *p* = 0.029; R^2^ = 0.21, *p* = 0.006; R^2^ = 0.30, *p* = 0.001; R^2^ = 0.18, *p* = 0.01) (Figure 9a). Among bacterial genera, the relative abundance of *Weissella* and *Pediococcus* genus bacteria were significant positive correlation with the content of lignin and acid detergent fiber. The relative abundance of Lactobacillus was significant positive correlation with the content of cellulose, hemicellulose and neutral detergent fiber (*p* < 0.05). It was reported that *Lactobacillus* sp., *Weissella* sp. and *Pediococcus* sp. were lactic acid-producing bacteria, which have a positive effect on the fermentation of silage [9,54]. In the meantime, the high proportion of *Lactobacillus* sp., *Weissella* sp. and *Pediococcus* sp. decreased the content of lignocellulose and improved the quality of silage. While the relative abundance of *Bacillus* sp. and *Paenibacillus* sp. showed a significant negative correlation with the content of silage chemical composition (*p* < 0.05). The fungal community compositions were significantly correlated with lignin and acid detergent fiber (R^2^ = 0.21, *p* = 0.003; R^2^ = 0.40, *p* = 0.001) (Figure 9b). Among fungal genera, the relative abundance of unclassified_fungi genus fungi was significantly positive correlated with the content of lignin and acid detergent fiber (*p* < 0.01), while *Monascus* and *Syncephalastrum* genus fungi were the opposite. *Monascus* sp. and *Syncephalastrum* sp. belong to mold. The study found that mold will lead to the loss of nutrients in silage, and the mycotoxins produced will pollute the feed and increase the possibility of animals suffering from fungal diseases [55].

The bacterial community compositions were significantly correlated with the activity of endoglucanase, β-glucosidase, neutral protease, and acid protease (R^2^ = 0.19, *p* = 0.009; R^2^ = 0.24, *p* = 0.002; R^2^ = 0.23, *p* = 0.003; R^2^ = 0.21, *p* = 0.005) (Figure 9c). Among bacterial genera, the relative abundance of *Lactobacillus*, *Bacillus* and *Paenibacillus* genus bacteria were significant positive correlation with the activity of endoglucanase, neutral protease, and acid protease. The relative abundance of *Weissella* sp. and *Pediococcus* sp. were significant positive correlation with the activity of β-glucosidase. *Lactobacillus*, *Weissella* and *Pediococcus* genus bacteria were dominant genera in silage fermentation, their abundance had important effects on the quality of feed [56]. This was confirmed by the positive correlation between their abundance and the activities of lignocellulose degrading enzymes. The fungal community compositions were significantly correlated with the activity of endoglucanase and neutral protease (R^2^ = 0.23, *p* = 0.005; R^2^ = 0.21, *p* = 0.009) (Figure 9d). Among fungal genera, the relative abundance of *Monascus* sp. was significant positive correlation with the activity of endoglucanase and neutral protease. It was found that *Monascus* sp. had fermentation function, so its abundance showed a positive correlation with enzyme activities [57], while the relative abundance of *Syncephalastrum* sp. showed significant negative correlation. There were few studies on the role of *Syncephalastrum* sp. in silage fermentation; Aamod et al. [58] found that *Syncephalastrum* sp. was a kind of spoilage fungi.

### 3.5. Co-Occurrence Network Characteristics of Bacterial and Fungal Communities

**Figure 10:**
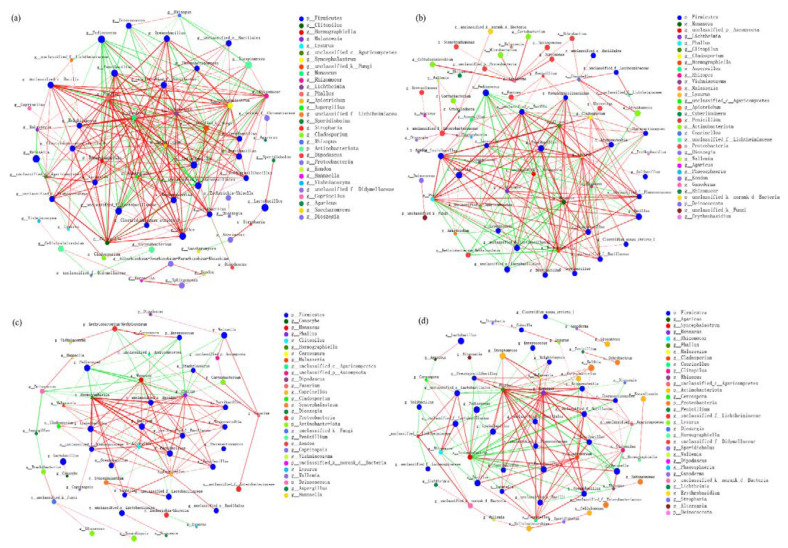
Co-occurrence network of fungal and bacterial communities during silage fermentation. (**a**) BP65, (**b**) BP80, (**c**) BP90, (**d**) BP100.

There were significant differences in microbial networks among different mixing ratios of *B. papyrifera* silage prepared with bran (Figure 10). The order of co-occurrence network complexity was BP65 > BP80 > BP100 > BP90. Compared with the co-occurrence network in BP100, more microbial species were found in BP65 and BP80. This indicated that there was a more complex and high diversity network in BP65. Each substrate had its own particularity, the protein content of *B. papyrifera* leaves was high, while the carbohydrate content of bran was rich [59]. After mixing, the substrate nutrition was more abundant, which could meet the growth and reproduction of more kinds of microorganisms. Meanwhile, greater resource availability and niche livability will also improve the stability of the microbial co-occurrence network [60].

There was no obvious relationship between the abundance of microbial genera and their importance in the network. The positive interactions in BP65 were 1.70 times greater than that of BP 100, and the number of edges were 1.54 times greater (Table 1). This indicated that there were less inter-species competitions in BP65. This may be due to the mixture of *B. papyrifera* and bran enriches the substrate nutrition of silage fermentation. More diversified resources can reduce competition in microbial communities, and provide more niches for the growth and reproduction of microbial groups [61].

There were different keystone species among different mixing ratios of *B. papyrifera* silage prepared with bran (Figure 10). The keystone species in BP100 were *Syncephalastrum* sp. and *Monascus* sp.; in BP90 were *Hormographiella* sp. and *Monascus* sp.; in BP80 were *Clitopilus* sp. and *Hormographiella* sp.; in BP65 were *Lichtheimia* sp. and *Phallus* sp. *Syncephalastrum* sp., which was usually considered as silage spoilage fungi, is known as a pathogenic microorganism [62]. The metabolites of *Monascus* sp. have been found to restrict the degradation of crude fibre [63]. The study found that *Lichtheimia* sp. could produce cellulases and hemicellulases [64], *Phallus* sp. was a member of manganese peroxidase producing fungi [65]. Compared with other mixing ratios, the positive interactions between key groups of microbial co-occurrence networks in BP65 were enhanced. This indicated that BP65 was better than other mixing ratios in improving the interspecific relationship of microbial groups, and was more conducive to the degradation of lignocellulose in silage fermentation.

## 4. Conclusions

The present study showed that co-ensiling of *B. papyrifera* with different proportions of wheat bran rich could significantly increase the degradation rate of hemicellulose, neutral detergent fiber, and the activities of filter paper, endoglucanase, acid protease, and neutral protease, especially in the ratio of 65:35. Mixing *B. papyrifera* with different proportions of bran had significant effects on the microbial community composition, metabolism function and co-occurrence network structure. Compared with monospecific *B. papyrifera* silage, adding bran increased the abundance of *Weissella* sp., bacterial fermentation potential and improved the relationships between different microbial groups in BP65. As the silage beneficial microbiota, the abundance of *Weissella* sp. was significantly increased after mixing *B. papyrifera* with bran in BP65. This may be an important factor to obtain better silage fermentation quality. Our results indicated that the contents of lignocellulose components in silage were closely related to the total abundance of LAB and the better interspecific relationships of microbial groups.

## Figures and Tables

**Table 1:** Microbial co-occurrence network properties in different samples.

Network Metrics	BP100	BP90	BP80	BP65
Number of nodes	69	57	66	59
Number of edges	166	120	215	256
Average connectivity	4.81	4.21	6.52	8.68
Average clustering coefficient	0.27	0.39	0.29	0.39
Positive interaction	111	89	146	189
Negative interaction	55	31	69	67

## Data Availability

All sequence data were deposited in the National Center for Biotechnology Information (NCBI) Sequence Read Archive (SRA) database.
